# Supplementary material for: Analysis of Early Cone Dysfunction in an In Vivo Model of Rod-Cone Dystrophy
Source: Int J Mol Sci. 2020 Aug 22;21(17):6055. doi: 10.3390/ijms21176055 (PMC7503557; doi:10.3390/ijms21176055)
Supplement: Supplementary file 1 [file ijms-21-06055-s001.pdf]

Supplementary material: Analysis of early cone dysfunction in an in vivo model of rod cone dystrophy. Hassall et al

Supplementary figure 1

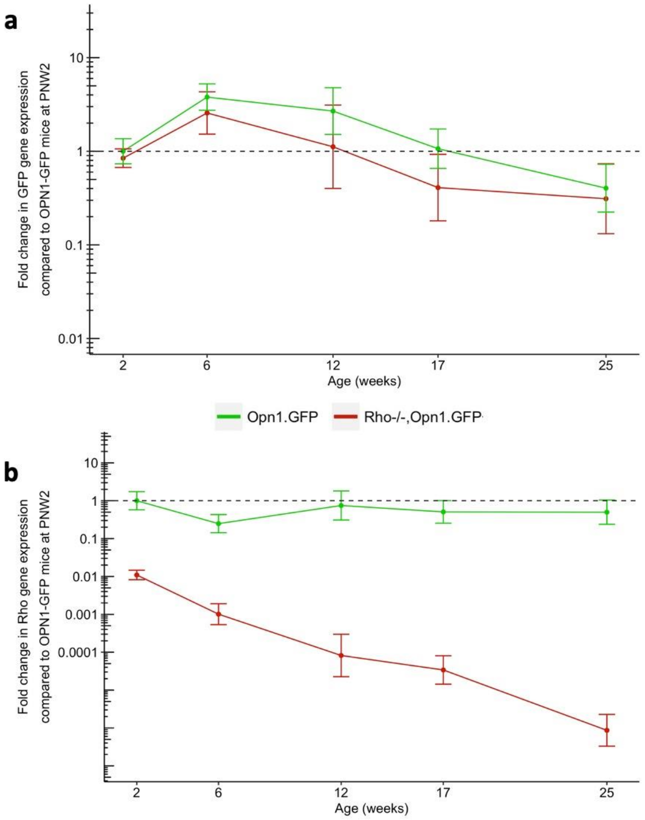

Supplementary figure 1: Additional qPCR gene expression data (a) GFP gene expression data over time (post-natal week, PNW, 2, 6, 12, 17 and 25) showing $2^{-\Delta\Delta Cq}$ values for *Rho*$^{-/-}$*OPN1-GFP* mice and *OPN1-GFP* mice. ΔΔCt values calculated by double normalising the test gene to the mean of *ActB* and *GFP* reference gene levels, then comparing each time point against *OPN1-GFP* mouse expression levels at PNW2 baseline. **(b)** Rhodopsin gene expression data for the same samples, noting the difference in y-axis magnitude. *All values are mean ± SEM. Tissue samples collected at PNW2 (OPN1-GFP n=8, Rho*$^{-/-}$*OPN1-GFP n=8), PNW6 (n=3, n=5), PNW12, (n=3, n=3), PNW17 (n=3, n=4), and PNW25 (n=3, n=3).*

Supplementary figure 2

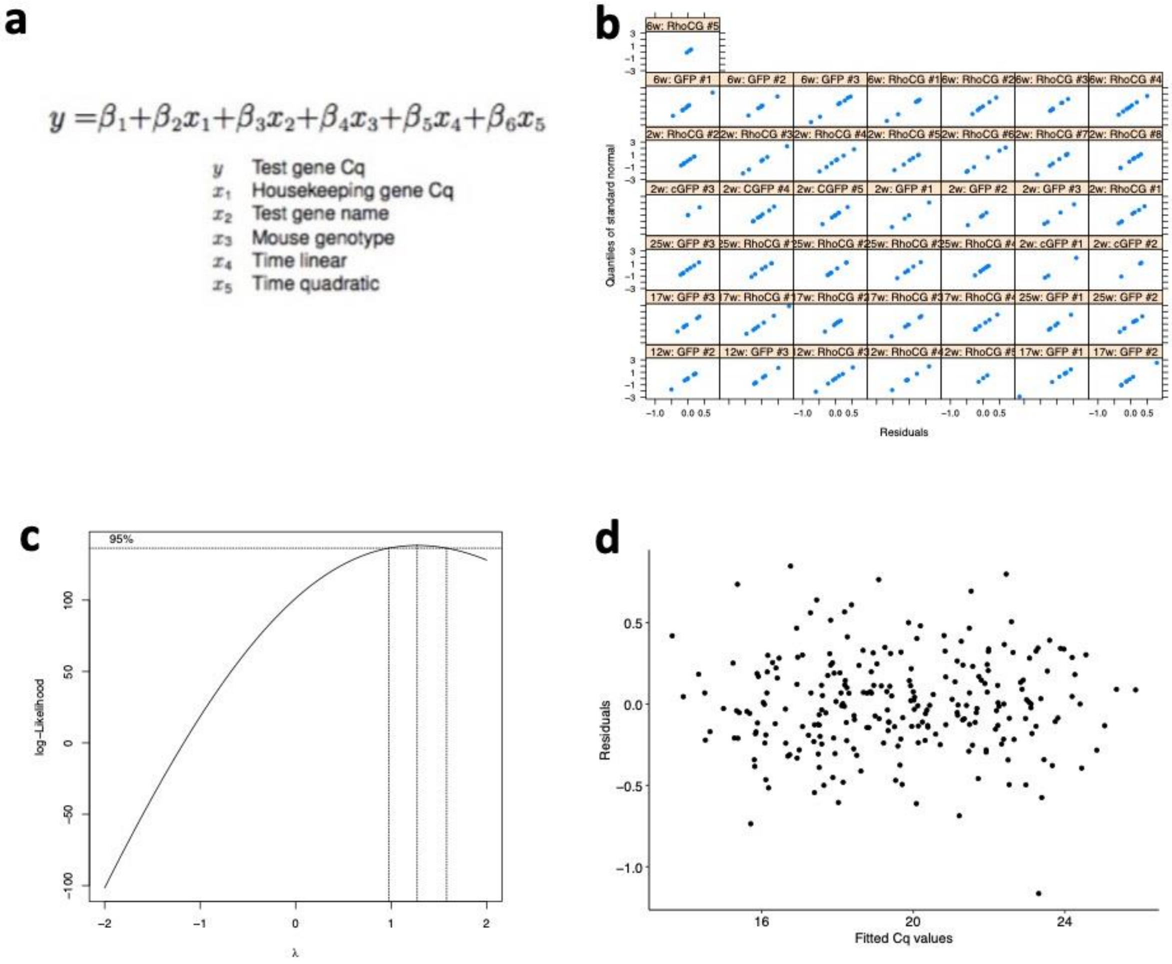

Supplementary figure 2: Hierarchical polynomial statistical model for qPCR data (a) Equation for statistical analysis denoting the uncorrected test gene Cq as the dependent variable, with housekeeping gene (mean of *Actb* and *GFP*) Cq as the first explanatory variable and interactions included between mouse genotype, the gene being tested, the linear time value and quadratic time value. **(b)** Regression model residuals showed some non-normality that was concentrated in a subset of mice, but no experimental basis was identified for exclusion. **(c)** The Box-Cox profile likelihood did not suggest any transformation. **(d)** A plot of fitted values against residuals showed homoscedasticity.

Supplementary table 1

outcome	Opn1mw	Opn1mw	Opn1sw	Opn1sw	Crx	Crx	Cnga3	Cnga3	ConeArr	ConeArr	Pde6h	Pde6h	Cngb3	Cngb3
Intercept	-12.061		-12.833		-14.303		-8.291		-11.311		-13.652		-8.915	
housekeeping	1.646		1.646		1.646		1.646		1.646		1.646		1.646	
GFP vs Rho-/-	0.382	0.501	-0.009	0.987	-0.106	0.852	0.083	0.882	-0.04	0.944	-0.041	0.942	-0.422	0.455
GFP PNW6 v baseline (PNW2)	1.08	0.162	0.395	0.604	1.464	0.061	2.219	0.006	0.492	0.517	0.367	0.639	1.789	0.024
GFP PNW12 v baseline	0.657	0.459	-0.317	0.720	0.56	0.528	1.468	0.121	0.573	0.515	0.757	0.396	1.452	0.108
GFP PNW17 v baseline	0.935	0.222	-0.736	0.333	-0.365	0.630	0.928	0.224	-0.069	0.927	0.68	0.382	0.918	0.231
GFP PNW25 v baseline	0.644	0.396	-0.674	0.385	-1.287	0.095	1.195	0.129	-0.248	0.742	0.285	0.706	0.308	0.683
vs Rho-/- PNW6	0.058	0.953	0.383	0.695	1.247	0.208	-0.145	0.883	0.918	0.359	0.649	0.519	0.381	0.699
vs Rho-/- PNW12	-0.253	0.826	1.195	0.308	2.037	0.084	-0.596	0.629	-0.807	0.489	-0.85	0.466	-0.864	0.459
vs Rho-/- PNW17	0.405	0.688	3.034	0.005	4.211	0.000	0.879	0.395	0.684	0.500	0.009	0.993	0.28	0.781
vs Rho-/- PNW25	1.616	0.116	4.441	0.000	5.091	0.000	1.578	0.132	2.067	0.047	1.712	0.098	0.823	0.416
Rho-/- PNW6 v baseline (PNW2)	1.138	0.085	0.778	0.229	2.711	0.000	2.074	0.003	1.41	0.035	1.017	0.132	2.17	0.002
Rho-/- PNW12 v baseline	0.404	0.588	0.878	0.254	2.597	0.001	0.871	0.290	-0.234	0.755	-0.094	0.903	0.588	0.444
Rho-/- PNW17 v baseline	1.34	0.054	2.298	0.002	3.846	0.000	1.807	0.015	0.614	0.364	0.689	0.312	1.198	0.083
Rho-/- PNW25 v baseline	2.259	0.002	3.767	0.000	3.804	0.000	2.773	0.000	1.818	0.010	1.996	0.006	1.131	0.100

Supplementary table 1: The statistical output of the polynomial model of cone cell qPCR data comparing *Rho$^{-/-}$*, *OPN1-GFP mice* to *OPN1-GFP* mice over time and rate of change.

A regression model, which included a quadratic polynomial relationship to time, was fitted to the qPCR data using the *nlme* package in R. Uncorrected test gene cycle quantification (Cq) was taken as the dependent variable, with housekeeping gene (*Actb*) Cq as the first explanatory variable and interactions included between mouse genotype, the gene being tested, and linear time as a factor (PNW2, PNW6, PNW12, PNW17, and PNW25). The diagnostic tests confirming a good model fit are provided **Supplementary Figure 1**. The results are also displayed graphically in **Figure 1** of the manuscript. Each individual gene is displayed in columns showing the magnitude of effect and p-value of each variable (fixed effect) in the hierarchical model. This table shows the relevant results of the polynomial model fitted to the repeated-measures qPCR dataset.

Supplementary figure 3 and table 2:

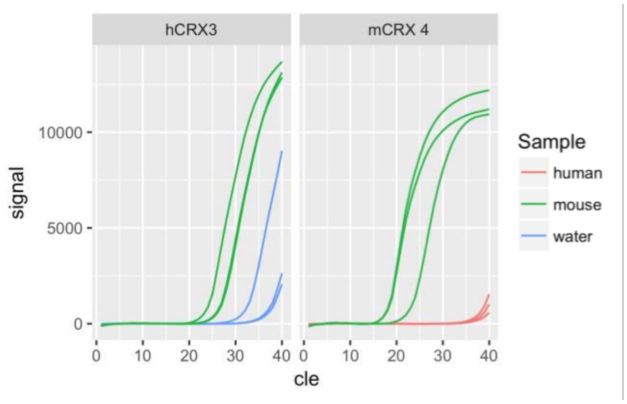

**Gene**	**Primer sequence**
Human CRX	FW-5’-AGGGCCGTCTCTGACCTCC-3’<br>RC-5’-GCTCGGAGACCCATAGGCG-3’
Mouse Crx	FW-5’-CACGTGAGGAGGTTGCTCTT-3’<br>RC-5’-GTAGAGGGTCTCGGGGATGT-3’

Supplementary table 2: Primer pairs for distinguishing between human and mouse orthologs of *CRX*. Mouse *Crx* mRNA is alternatively spliced as isoform 1 (NM_007770.4) or isoform 2 (NM_001113330.1); however, the coding sequence of both isoforms share 87.5% homology with human *CRX* coding sequence (NM_000554.6). In both instances, the nucleotide differences are sparsely spread across the transcript. In an attempt to differentiate between exogenous human transgene sequence and endogenous murine nucleotide sequence, eight qPCR primer pairs were compared.

Supplementary table 3:

Gene	Direction	Sequence (5'-3')
Opn1.GFP	FW	CACCTACGGCAAGCTGAC
	RC	CTTGTAGTTGCCGTCGTC
Crx	FW	CACGTGAGGAGGTTGCTCTT
	RC	GTAGAGGGTCTCGGGGATGT
Cnga3	FW	TAGACATGCTGGTTCGAGC
	RC	GTTGGTCCTTGTCTCTGTG
Cngb3	FW	AGCCAGCTCTCAGACTGCA
	RC	ACAGTTCCAGTTATACGCA
Arr3	FW	GTGGATGATGTGGACACTG
	RC	CTGGATGCTGGTGGGTTC
Pde6h	FW	GAGTCCTCCAGCACCAAG
	RC	TCCCGAACTGAGCAAGCT
Opn1sw	FW	ACAGTCTTCATCGCCA
	RC	CAAGTAGCCAGGACCAC
Opn1mw	FW	CAGCAACAGCACCAAAGGTC
	RC	GCAACTGCCAAGTTCACCAG
Rho	FW	TGCTCAACTTGGCCGT
	RC	GCGGAAGTTGCTCATCG
ActB	FW	AGCCATGTACGTAGCCA
	RC	GAAGCTGTAGCCACGCT

Supplementary table 3: Primer pairs for the cone phototransduction cascade gene qPCR. The optimised primer pairs used in the qPCR experiment to characterise the changes in cone gene expression over time in *Rho*$^{-/-}$, *OPN1-GFP* mice, compared to the *OPN1-GFP* mice as a reference.

Supplementary figure 4

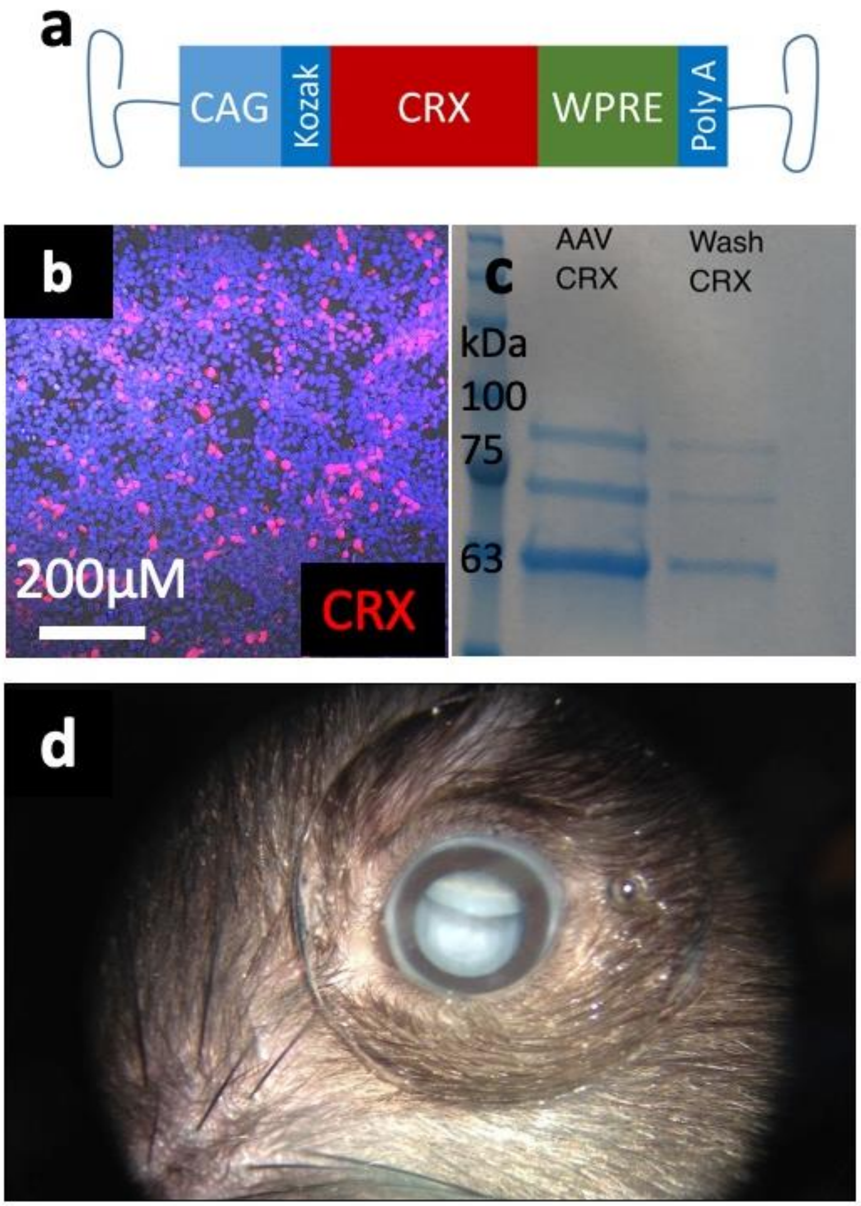

Supplementary figure 4: Development and delivery of AAV (a) Representation of the rAAV2/8.CAG.CRX.WPRE.pA vector structure. **(b)** The rAAV constructs was validated by transfecting HEK cells, culturing, and then staining fixed cells for CRX protein to confirm transgenic protein production under control of the mammalian promoter. **(c)** EZ Blue stained SDS gel showing electrophoretically separated protein bands of a protein reference ladder, concentrated AAV preparation and AAV wash. The three blue bands correspond to the three subunits of the AAV capsid (87, 73 and 62kDa). **(d)** An example of subretinal delivery of rAAV.CRX vector into *Rho*$^{-/-}$, *OPN1-GFP* mice, with the intended retinal detachment visible superiorly between 10 O'clock and 2 O'clock.

Supplementary figure 5

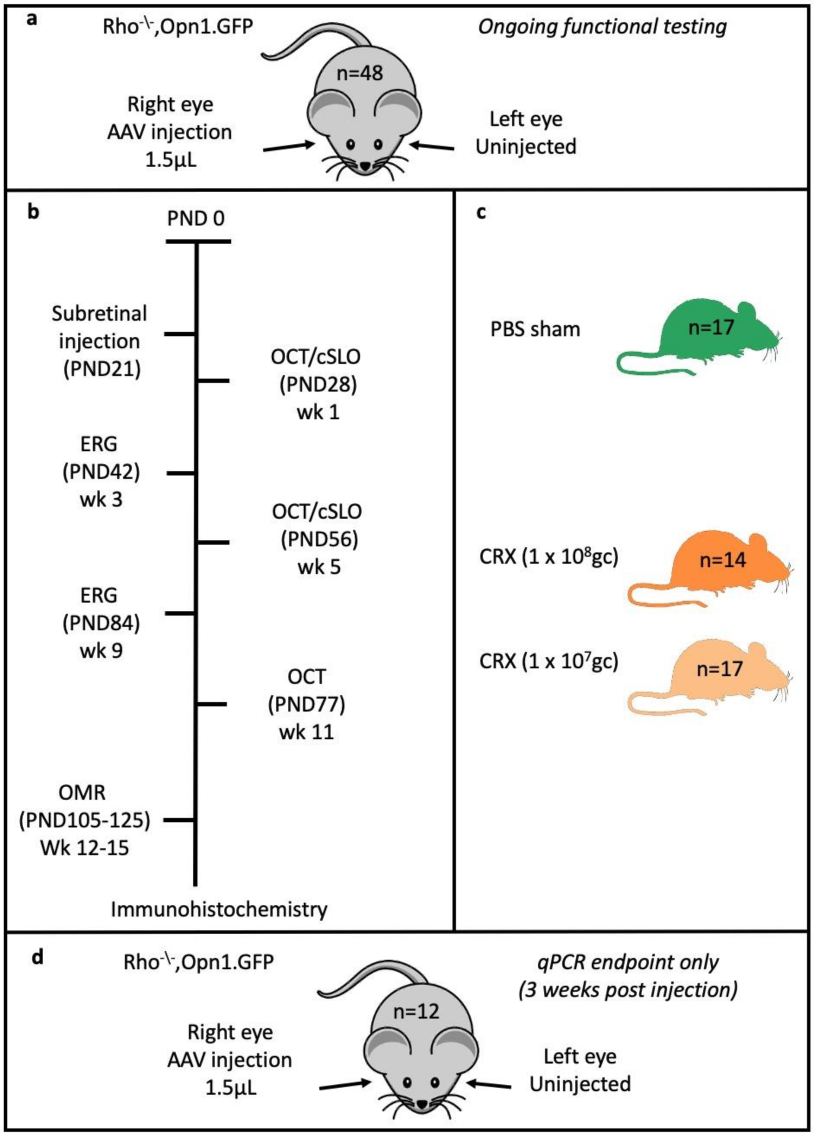

Supplementary figure 5: Cohorts and study design. (a) The study consisted of 48 *Rho*$^{-/-}$, *OPN1-GFP* mice receiving subretinal injections of rAAV.CRX or PBS into one eye only; the fellow eye remained an uninjected control. **(b)** This cohort was followed over 14 weeks following injection (some were sacrificed 7 weeks post injection for IHC; n=6) with ERG, OCT, cSLO and OMR, before being sacrificed for IHC. **(c)** Mice were allocated to one of two treatment groups or sham. **(d)** An additional 12 *Rho*$^{-/-}$, *OPN1-GFP* mice received subretinal injections of rAAV.CRX or sham for separate qPCR analysis 3 weeks post injection.
